# Court Procedures for Handling Intoxicated Drivers

**Published:** 2001

**Authors:** Robert B. Voas, Deborah A. Fisher

**Affiliations:** Robert B. Voas, Ph.D., is a senior research scientist and Deborah A. Fisher, Ph.D., is an associate research scientist at the Pacific Institute for Research and Evaluation, Calverton, Maryland

**Keywords:** court ruling, sanction, drinking and driving, impaired driver, rehabilitation, drug court, license suspension, ignition interlock device, electronic monitoring of offenders, deterrence of AODU (alcohol or other drug [AOD] use, abuse, and dependence), AOD education

## Abstract

The courts have implemented numerous approaches to reduce the probability of recidivism among people apprehended for or convicted of driving while intoxicated. Although traditional punitive sanctions, such as fines and incarceration, are commonly used, they have not eliminated drinking and driving in the United States. Consequently, the court system has developed additional sanctioning procedures that show promise. For example, rehabilitative programs (e.g., alcohol education and alcoholism treatment) can reduce recidivism, at least marginally. These programs appear to be more effective when combined with license suspension. In addition to license suspension, several alternative methods for limiting driving opportunities of offenders have proven effective, including impounding offenders’ vehicles or license plates, installing ignition interlocks, and requiring electronic home monitoring or house arrest. Effective court monitoring is a critical component in supporting recovery and compelling offenders to participate in rehabilitation programs. This role of the courts in monitoring offenders will likely increase as the use of intrusive, alternative sanctions grows.

During the past two decades, the percentage of U.S. highway fatalities that can be classified as alcohol related (i.e., those involving alcohol-positive road users, such as drivers, bicyclists, or pedestrians) has fallen from 57 to 38 percent (National Highway Traffic Safety Administration [NHTSA] 1999). The reasons for this extensive reduction in alcohol-related highway fatalities are not fully understood. It is clear, however, that this decline coincides with a significant increase in the number and severity of drunk-driving laws. Such laws serve two major functions. First, they help prevent impaired driving by the public (i.e., exert a general deterrent effect) by increasing the perceived risk of arrest and sanctioning. Second, they reduce the likelihood of recidivism (i.e., exert a specific deterrent effect) by imposing sanctions on people who are apprehended for driving while intoxicated (DWI). This latter deterrent primarily concerns the courts, which handle DWI offenders.

The relative importance of specific versus general deterrence is unclear and somewhat controversial, because estimates vary widely regarding the proportion of drinking drivers involved in fatal crashes who have been previously convicted of DWI (i.e., who are repeat offenders). For example, data from the Fatality Analysis Reporting System (FARS), which includes information on arrests in the 3 years preceding the crash, have suggested that 11 percent of offenders are repeat offenders (Hedlund 1995). In contrast, data based on State record systems that include 7 to 10 years of arrest information have indicated that the proportion of repeat offenders is more than 30 percent (Simpson et al. 1996). Thus, for up to one-third of drinking drivers involved in fatal crashes, the courts have had a previous opportunity to intervene and reduce the risk of recidivism by implementing court programs.

This article provides an overview of the court procedures currently used to handle DWI offenders. The article first reviews some general considerations and historical developments in identifying and sanctioning DWI offenders, followed by a discussion of various types of sanctions used. Finally, the article explores the judicial process for adjudicating DWI offenses and the various stages in that process during which interventions can occur.

## DWI Offenses and Recidivism

A large proportion of motorists whose driving is impaired by alcohol go undetected, as evidenced by the fact that at least two-thirds of the most serious (i.e., fatal) alcohol-involved crashes are caused by drinking drivers who have never before been apprehended by the police. This finding is confirmed by roadside breath-test surveys that determine the number of drinking drivers on U.S. roadways (Voas et al. 1998). Based on such surveys, estimates of the number of times a person drives drunk before being arrested have ranged from 300 (Voas and Hause 1987) to 2,000 (Borkenstein 1975). Thus, both DWI arrests and crashes are infrequent occurrences for intoxicated drivers. This observation has two implications for the courts in handling DWI offenders. First, few drivers coming before the courts for the first time are actually first-time offenders. Most have driven under the influence many times without being apprehended. Second, many people who drive while impaired do not get caught and arrested or are not involved in crashes.

As a result of the low probability of being apprehended for DWI, many offenders who come before the court will never be arrested again (i.e., will not be considered recidivists), regardless of the action the court takes or whether the offender continues drinking and driving. Another factor influencing recidivism rates is the fact that spontaneous changes in impaired driving behavior occur in many people’s lives. Thus, many people who drink and drive at one point in their lives may subsequently change their drinking or driving behavior and avoid driving while impaired without undergoing any formal intervention from either health or legal agencies.^1^ Finally, the number of recidivists obviously depends on the level of enforcement in the community as well as the effectiveness of court-sanctioning programs.

Some information exists regarding the recidivism rates of people convicted of DWI. For example, data from the State of Michigan, which maintains a 10-year record of DWI convictions in its drivers file, indicate that 38 percent of first-time offenders commit a second offense (Michigan Office of Public Safety 1998). In other words, approximately two-thirds of first-time DWI offenders do not commit or are not apprehended for a subsequent offense. Unfortunately, it is difficult to determine what portions of this “success” are attributable to court actions, to spontaneous behavior changes that would have occurred independent of the arrest and conviction for DWI, or to the low probability of being apprehended.

The fact that more than one-third of first-time DWI offenders are re-arrested has led to concerns about “hard-core” drinking drivers who are so frequently impaired when driving that they have a record of multiple convictions despite the generally low apprehension rates. These drivers are at especially high risk of being involved in alcohol-related crashes (Simpson et al. 1996). Based on their analyses, Simpson and colleagues (1996) suggested a definition for hard-core drinking drivers that includes all multiple offenders and first-time offenders who have high blood alcohol concentrations (BACs) (i.e., 0.15 percent or more) when arrested. A high BAC generally is viewed as an indication that the offender has established a pattern of heavy drinking over a substantial period of time, resulting in sufficient tolerance to be able to reach BACs of 0.15 percent or higher. This definition has been adopted by several safety organizations and Government agencies, including The Century Council (2000), Mothers Against Drunk Driving (MADD) (Voas et al. 1999), and the National Transportation Safety Board (2000). Approximately one-half of first-time DWI offenders have BACs of at least 0.15 percent when arrested. When these offenders are added to the number of offenders with more than one conviction, more than two-thirds of all DWI offenders who come before the court can be classified as hard-core drinking drivers.

Legislative agencies have focused on hard-core-drinking drivers, particularly repeat offenders, for a long time. Following the establishment of the Department of Transportation in 1967, the Highway Safety Bureau (which later became the NHTSA) issued a report on Alcohol and Highway Safety that suggested “problem drinkers”^2^ comprised as much as two-thirds of the drinking drivers involved in fatal crashes (U.S. Department of Transportation 1968). As a result of this analysis, the Federal Government funded 35 Alcohol Safety Action Projects (ASAPs) in communities across the Nation. At the time, courts principally used fines, license suspension, and incarceration as sanctions for drinking drivers. The ASAPs’ focus on problem drinkers, however, stimulated the courts to add alcoholism treatment and alcohol education programs to the mix of sanctions used with DWI offenders (Stewart and Ellingstad 1989). Although the ASAPs were terminated when Federal funding was discontinued, the use of rehabilitation options by the courts in the ASAP communities continued and spread to most traffic courts, at least in part because the offenders, not the Government, pay the costs of treatment services.

Interest in hard-core drinking drivers has increased again in recent years, in part because of the controversy over legislation to reduce the legal BAC limit from 0.10 to 0.08 percent. Groups opposing such laws have argued that legislation could be directed more profitably at the problem drinkers arrested with high BACs who are at greater risk of being involved in crashes than are the heavy consuming “social drinkers,” who are likely to be arrested with BACs between 0.08 and 0.10 percent. Because of this political debate, in 1998 Congress added a section to the Transportation Equity Act for the 21st Century (TEA–21) requiring States to strengthen their sanctions for second-time DWI offenders or face a transfer of highway construction funds to safety purposes.

Another development that increased public and legislative attention to the impaired-driving issue was the formation of activist groups, such as MADD, in the early 1980s. The advocacy efforts of these groups resulted in legislation that significantly enhanced the severity of sanctions for DWI and strengthened enforcement of impaired driving laws, thereby increasing the flow of offenders into the courts (McCarthy and Harvey 1989). MADD also initiated efforts to identify limitations of the adjudication process. For example, the group organized a court-monitoring program that revealed the extent to which offenders often were able to avoid the more severe sanctions. In particular, the courts frequently failed to impose the full period of license suspension provided by the law, even though extensive research indicated that license action was the most effective sanction for reducing recidivism and crash involvement of DWI offenders (Nichols and Ross 1990; Peck et al. 1985).

The inconsistent application of license sanctions by the criminal justice system encouraged activists to push for the passage of administrative license revocation (ALR) laws. These laws empower the State departments of motor vehicles (DMVs) to suspend, at the time of arrest, the drivers’ licenses of those people who are apprehended with BACs above the legal limit. (This license suspension takes place prior to the trial and is generally independent of the court disposition.) ALR laws, which currently exist in 40 of the 50 States, are associated with reduced proportions of drinking drivers in fatal crashes (Zador et al. 1988; Voas et al. 2000) and have been highly successful in increasing the number of DWI offenders whose licenses are suspended. However, States are experiencing difficulties in adequately enforcing those suspensions: as many as 75 percent of drivers suspended for DWI continue to drive (Nichols and Ross 1990). As a result, both public and legislative attention is focusing increasingly on court sanctions that prevent DWI recidivism and driving-while-suspended offenses through actions against the offender’s vehicle, such as impoundment or confiscation. These and other court sanctions currently used with DWI offenders are discussed in the following section.

## Sanctions Used With DWI Offenders

As mentioned previously, any sanction can have a general deterrent effect as well as a specific deterrent effect. The discussion in this article focuses on the impact of sanctions in preventing people who have been apprehended for DWI from repeating their offense (i.e., on specific deterrence strategies). The generation of a general deterrence effect—that is, dissuading the driving public at large from ever drinking and driving—is of equal or possibly even greater importance, however, and each sanction must be judged on its merits for both general and specific deterrence. This requirement complicates the evaluation of court remedies applied to DWI offenders. Space does not permit the assessment of the general deterrence value of sanctions in this review, however, and the reader is referred to articles by Voas (2000), Hingson (1996), and Nichols and Ross (1990) for a broader discussion of the general deterrence effects of various sanctions.

Court sanctions for DWI offenders fall into four categories based on different concepts of why people drink and drive and how recidivism can be prevented:

- *Penalties to produce deterrence.* The use of these sanctions (e.g., fines and incarceration) is based on the assumption that drinking and driving occurs because the driver is not motivated to change his or her behavior and perhaps accept inconveniences (e.g., relying on a designated driver or taxi) to avoid drunk driving. In these cases, punishment (or the threat of punishment) might favorably influence future decision-making about drinking and driving.
- *Education programs.* These sanctions assume that the driver committed the DWI offense because of a lack of knowledge about the following: drinking-and-driving laws, the effects of alcohol on driving, and ways to avoid drinking and driving. Education programs, together with alcoholism treatment programs, are classified as rehabilitative approaches.
- *Alcoholism treatment programs.* The use of these sanctions is based on the premise that many DWI offenders abuse or are dependent on alcohol and must recover from their uncontrolled pattern of alcohol consumption in order to avoid impaired driving.
- *Incapacitating sanctions.* These measures, which include license suspension and vehicle actions (e.g., vehicle impoundment), are designed to protect the public (at least for the duration of the sanction) by making it impossible for the offender to drink and drive, regardless of the reason for the original offense.

Many DWI sanctions fulfill more than one of these objectives, because the specific deterrent, the rehabilitative approaches, and the incapacitating effects of sanctions often cannot be separated. For example, both incarceration and the installation of devices that prevent an intoxicated driver from starting a vehicle (i.e., ignition interlocks) can have an immediate effect in protecting the public by restricting or eliminating driving. Simultaneously, these measures are expected to be aversive and to deter recidivism. Similarly, although treatment and education programs may serve primarily as a means to rehabilitate offenders, they also may be experienced by offenders as unpleasant and costly and thus have a specific deterrent effect as well.

### Deterrence

All court sanctions have a potential deterrent effect, because they force the offender to engage in activities that he or she would normally avoid. Traditionally, however, fines and jail sentences have been the primary means of creating deterrence.

#### Fines

Despite the widespread use of fines in penalizing DWI offenders, little research has been conducted regarding their general or specific deterrent effect in the United States. Some evidence from other western, industrialized countries (e.g., Australia and Sweden) indicates that fines can be effective deterrents, particularly the relatively high fines instituted in Scandinavian countries that are based on the offender’s income and the seriousness of the offense (e.g., the offender’s BAC at the time of the incident) (Nichols and Ross 1990). In the United States, however, many fines either are not collected or can be paid in increments over a long period and thus do not place a substantial financial burden on the offender. Furthermore, courts frequently waive fines in order to enable the offender to afford to pay for a required treatment program. Consequently, fines are not often implemented to maximize deterrence (i.e., as a swift, certain, and substantial penalty), making it difficult to evaluate their effect. Moreover, an assessment of the deterrent effect of fines is complicated by the additional monetary costs associated with a DWI conviction, including lawyers’ fees, increased automobile insurance rates, license reinstatement fees, and possible costs of court-mandated treatment.

In addition to their punitive function and potential for reducing recidivism, fines fund the DWI criminal justice system in some jurisdictions. The NHTSA (1983) has urged States to develop “self-sufficient” alcohol safety programs. The State of New York has come closest to meeting this goal by adopting a system whereby DWI fines that go to the State are returned to counties that establish “Stop DWI” programs with a full-time manager. Fines also can be used to support public information and enforcement efforts (e.g., sobriety checkpoints).

#### Incarceration

Only limited evidence suggests that jail sentences have a general deterrent effect and reduce drunk driving by the public (Voas 1986; Nichols and Ross 1990; NHTSA and the National Institute on Alcohol Abuse and Alcoholism [NIAAA] 1996). Moreover, little evidence indicates that this sanction has a specific deterrent effect, making its usefulness questionable. In addition, limited jail space and the cost of incarceration constrain the use of jail sentences (Voas 1985). Nevertheless, the authority to incarcerate an offender ultimately is the basis of the court’s power to enforce all of its other sanctions (e.g., probation requirements and referrals to treatment programs). Thus, although the effectiveness of jail sentences is doubtful, the desire to avoid jail is an essential incentive for offenders to comply with sanctions that appear to be more effective, such as treatment interventions (NHTSA and NIAAA 1996).

Jail sentences also can provide an opportunity to place DWI offenders into residential treatment programs. For example, Prince George’s County, Maryland, has implemented a 28-day DWI detention center program for repeat offenders in which participants undergo intensive assessment and receive group therapy in the evenings after returning from work release. This program reduced repeat-offender recidivism by approximately 75 percent (Voas and Tippetts 1990). The passage of a law requiring a mandatory 2-day jail term for first-time DWI offenders in Ohio provided an opportunity to develop a 48-hour residential “Weekend Intervention Program” (WIP) that met the State’s jail requirements but also provided for intensive assessment of first-time DWI offenders (Siegal 1985). Final disposition of the case was postponed until the detailed referral plan resulting from this assessment could be submitted to the court for use in sentencing offenders. This program appeared to reduce recidivism when the judge made adherence to the referral plan a condition of probation (Siegal 1985), and the program has been duplicated in numerous areas throughout the country.

### Rehabilitation

The use of rehabilitative or “remedial” sanctions is based on the assumption that for many drivers, DWI offenses result from personal risk factors, such as lack of knowledge about alcohol’s effects or the presence of alcohol-use disorders (i.e., alcohol abuse or dependence). To change those risk factors, the offenders are mandated to participate in education or alcoholism treatment programs.

#### Education Programs

Education programs provide information on important alcohol-related issues, such as alcohol’s effects on driving performance, the relationship between rate of consumption and BACs, and the nature of DWI laws. In addition, these programs frequently include skills training to help offenders choose and implement plans to avoid drinking and driving. These programs, which are aimed primarily at first-time DWI offenders who have been classified as “social drinkers,” generally are delivered in a classroom setting over 10 to 14 hours.

Evaluations of such programs have yielded mixed results, with the extent of the impact frequently inversely related to the quality of the evaluation design (Stewart and Ellingstad 1989; Wells-Parker et al. 1995). For example, early evaluations that found relatively great benefits from education programs suffered from the comparison of nonequivalent groups. Thus, participants in education programs were not randomly assigned to those programs, but elected to do so to avoid license suspension; as a result, the studies compared these fully licensed drivers with suspended drivers. The opportunities to conduct random assignment studies within the court setting, however, have been limited by fairness and equal treatment considerations. Even when such opportunities have existed, they have frequently yielded conflicting results (for an example of such conflicting results obtained in the same State, see Reis 1983 and Stewart et al. 1987).

One “educational” effort being applied throughout the country to both first-time and repeat offenders is mandated attendance at Victim Impact Panels (VIPs). These panels are one-time, 2-hour education programs in which victims of drunk-driving crashes or their relatives describe their injuries and the effect the crash has had on them. Two evaluations of VIP programs have been published. Fors and Rojeck (1999) reported evidence for the programs’ effectiveness in reducing recidivism; however, a study by Shinar and Compton (1995) produced equivocal results.

#### Treatment Programs

Therapeutic programs strive to reduce impaired driving by promoting recovery from alcohol abuse and dependence. These interventions require a more extensive and intensive form of remediation than can be achieved with education and counseling. Historically, approximately one-third of DWI offenders have been classified as “problem drinkers” based on analyses of their driving records and on brief assessment instruments, such as the Michigan Alcoholism Screening Test (MAST) or the Mortimer-Filkins structured interview (Nichols et al. 1978). These offenders typically have been assigned to 3- to 6-month treatment programs (Nichols et al. 1978). The remaining two-thirds of first-time offenders have been considered “social drinkers” who are candidates for education programs.

To motivate DWI offenders to participate in longer therapeutic programs, participants initially were allowed to retain their driving privileges. When the recidivism rate of offenders in treatment programs was compared with that of offenders whose driving privileges had been fully suspended, however, the latter group was involved in fewer subsequent crashes (Hagen et al. 1980; Sadler et al. 1991). This difference appears to result principally from the fact that suspension reduces the risk of both alcohol-related and non-alcohol-related crashes, whereas treatment primarily reduces alcohol-related crashes (McKnight and Voas 1991). Although the effects on crash involvement associated with suspension are more extensive than those associated with treatment, the former tend to be shorter in duration, lasting only as long as the suspension is in effect. In contrast, treatment may have carry-over effects (e.g., reductions in heavy drinking) that extend well beyond the period of intervention.

More recently, with the passage of ALR laws, all DWI offenders tend to receive suspensions regardless of whether they participate in treatment programs. Under these conditions, the combination of suspension with treatment has been more effective in reducing recidivism than suspension or treatment alone. For example, DeYoung (1997) found that suspended second-time offenders who participated in an 18-month treatment and monitoring program had recidivism rates approximately 30 percent lower than did offenders who only received suspensions.

In general, the evaluation of treatment and education programs for DWI offenders has proven difficult. (For a recent review of the methodological challenges inherent in conducting research related to the broader effectiveness of alcohol and other drug treatment as well as the strategies for addressing them, see Dennis et al. 2000.) Among the factors contributing to the methodological shortcomings of studies are the following:

- Judges are guided by fairness and equal treatment considerations when making their decisions. As a result, they generally do not assign offenders randomly to various court programs, which can result in non-comparable groups of offenders.
- Researchers and judges generally have a tendency to treat DWI offenders as a homogeneous group and ignore important variations among them. However, researchers have identified 5 to 10 groups of offenders with significantly different characteristics. These differences can make it harder to detect specific program effects.
- Truly “untreated” control groups to compare with treated offenders are usually lacking, because offenders in control groups generally receive a set of traditional sanctions, which may be more salient than the treatment being evaluated.
- Researchers often use relatively weak measures for evaluating treatment and education programs. For example, some studies use DWI recidivism and crash involvement as outcome measures, both of which are based on rare events and therefore are relatively insensitive. Other studies rely on self-reported attitude and knowledge items, which are subject to recall and other biases and, consequently, may be of limited validity for predicting recidivism.

In a landmark study, Wells-Parker and colleagues (1995) conducted a meta-analysis of 215 studies of rehabilitative interventions for DWI offenders reported between 1955 and 1992. Interventions fell into seven categories: education, psychotherapy or counseling, traditional alcoholism treatment, Alcoholics Anonymous, treatment with the medication disulfiram (Antabuse^®^),^3^ contact probation, and combined interventions (i.e., interventions that included two or more distinct and independent modalities). Interventions involving education only lasted an average of 10 hours over 5 weeks, whereas combined interventions lasted an average of 36 hours over 32 weeks. Given the limitations noted above, it is not surprising that the majority of studies analyzed were considered methodologically weak. However, the investigators found that among the studies rated as strongest methodologically, remedial programs were associated with a 7- to 9-percent reduction in recidivism. Combinations of modalities—in particular those including education, psychotherapy or counseling, and followup contact or probation—were more effective than other intervention types. A recent review of meta-analyses conducted on alcohol and other drug treatment research (Wilson 2000) points out some problems with the analytic techniques used in the study by Wells-Parker and colleagues (1995); however, to date, it is the only attempt to quantitatively synthesize the research on remedial programs for DWI offenders.

Reductions in recidivism of 7 to 9 percent may not appear to be substantial. However, about 1.5 million DWI arrests occur each year (Federal Bureau of Investigation 2000 online), and most of the offenders are convicted and required to attend an education or treatment program. With these high numbers, a 9-percent reduction in recidivism can result in a major reduction in repeat DWI offenses. Further, recidivism only reflects the potential benefits of treatment and other sanctions to highway safety. However, reductions in heavy drinking produced by treatment programs also can reduce alcohol-related violence, falls and other traumatic injuries, and other medical and social consequences of excessive alcohol use. The potential effect of these non-highway safety benefits was demonstrated by Smart and Mann (1993), who found an association between the regional availability of treatment programs and lower liver cirrhosis rates in Canada.

### Incapacitation

#### Vehicle Impoundment

As previously noted, the passage of ALR laws has increased the use of license suspensions and has largely taken the right to impose this sentence out of the hands of the courts. However, States are encountering a significant problem in enforcing license suspensions, as shown by the fact that 50 to 75 percent of DWI offenders report that they have driven while suspended (Nichols and Ross 1990). In addition, most offenders do not reinstate their licenses when they become eligible to do so. For example, the reinstatement rate in California— which has an estimated 1 million suspended drivers—is only 16 percent (Tashima and Helander 1999). This failure results, in part, from the high cost of insurance and from the State fees and regulations surrounding reinstatement. Regardless of the reasons, however, this failure to reinstate their licenses suggests that those offenders are not greatly concerned about being apprehended and convicted of driving while suspended.

As a result, courts have increasingly turned to measures targeting the vehicles (and, in some cases, the registration or vehicle tags) of DWI offenders as a means of incapacitating these high-risk drivers. For example, in 1993 Ohio implemented a 3- to 6-month vehicle impoundment/immobilization sanction for repeat DWI offenders. This program reduced recidivism among fully suspended offenders (who should not have been driving in the first place) by as much as 50 percent while the vehicle was held by the State. A smaller (i.e., 20 percent) reduction in recidivism continued for up to 1 year after the vehicle was returned. Vehicle impoundment was also an effective method of reducing impaired driving in California (Voas and DeYoung in press). One problem associated with vehicle impoundments is that many vehicles are not owned by the offenders but by other persons (e.g., the spouse of the offender). In these cases the vehicles are usually released if the owner pays the towing and storage costs and signs an agreement not to allow the offender to use the car. Despite this complication, use of this sanction is expected to increase as the Federal Government has made impoundment or installation of an interlock a requirement for sanctioning second DWI offenders in the TEA–21 legislation.

#### Ignition Interlocks

Alcohol safety interlocks are also widely used to control the driving of DWI offenders. These devices require the driver to take a breath test before starting the car and will prevent vehicle ignition if the operator has a BAC higher than 0.025 percent. Interlock devices are highly effective in preventing drinking and driving, thereby reducing DWI recidivism by 50 to 90 percent (Coben and Larkin 1999; Voas et al. 1999) while they remain on the vehicle. Once the units are removed and the offender’s license is reinstated, however, recidivism risk returns to the same level as that of offenders who have not participated in interlock programs.

Eighty percent of States have passed specific legislation allowing the use of interlock devices; however, fewer than 10 percent of offenders who are offered a choice between installing such a device or receiving a license suspension elect to enter an interlock program (Voas et al. 1999). Thus, the majority of DWI offenders apparently prefer to drive while suspended rather than install such units. Currently, both the courts and the State DMVs manage interlock programs. The courts, however, with their ability to motivate compliance through the threat of other sanctions (e.g., incarceration) will increasingly be called upon to manage these programs in order to achieve higher levels of participation by DWI offenders.

#### License Plate Impoundment

In 1988 Minnesota enacted a law that provides for impounding the license plate of DWI offenders who have received either two prior DWI convictions in the preceding 5 years or three or more convictions in the preceding 10 years. When the law first took effect, drivers were expected to surrender their license plates to the court for destruction; however, less than 5 percent of the offenders complied. As a result, the law was subsequently changed to provide for administrative impoundment and destruction of the plates by the arresting officer or, if the officer did not, by the Department of Public Safety. When the law was administratively enforced, it significantly reduced recidivism (Rodgers 1994). Moreover, the law appeared more effective in reducing recidivism when the impoundment occurred at the time of arrest rather than later.

#### House Arrest and Electronic Home Monitoring

A relatively recent type of sanction is placing the offender under house arrest (while allowing him or her to go to work or attend other court-approved activities), which is enforced through electronic monitoring (e.g., electronic bracelets). Electronic house arrest generally has been considered an alternative to jail. If, however, the duration of the house arrest were equal to the corresponding jail sentence, such a sanction would be viewed as significantly less severe. Therefore, house arrest periods often are relatively lengthy (i.e., 3 to 6 months) compared with jail sentences given for the same offenses. Such periods can be considered an incapacitating sanction: they prevent recreational and nighttime driving, because the offender is confined to the home. An evaluation of a house arrest program for DWI offenders in Los Angeles County (where the average confinement was 88 days) found that the program reduced recidivism by approximately one-third (Jones et al. 1996).

## Court Procedures Used for Imposing DWI Sanctions

The handling of DWI cases in the court system has several characteristics that influence how and at what stage in the judicial process DWI sanctions, such as those discussed in the previous section, are imposed. For example, most DWI cases are settled through plea bargains (i.e., without formal trials). Accordingly, the courts have developed various shortcut procedures involving pretrial and presentencing programs. Also, DWI cases tend to be the least interesting and least challenging cases for the judiciary and, therefore, are frequently assigned to junior members of the court. Furthermore, because DWI tends to be the most frequent criminal offense, case loads are high and judges are under pressure to avoid jury trials. As a result, judges try to limit hearings to no more than one per offender. The procedures used by the courts to implement the various sanctioning programs have received little research attention. The following sections summarize some of those procedures and their effect on sanctions.

### Juvenile Courts

Offenders younger than age 18 can be tried in either juvenile or adult court. The use of juvenile courts has both advantages and disadvantages. For example, juvenile courts can play an important sanctioning role, because they have the power to require parental involvement in remedial programs. At the same time, however, safety activists have been concerned about the use of juvenile courts, because the court records are sealed, thereby making it difficult to determine whether an offender has received a sanction. Moreover, DWI convictions in juvenile court frequently are not reported to the DMV; as a result, the public is not protected against repeat offenses by suspension of the youth’s license. Consequently, most localities try underage DWI offenders in regular traffic courts. Similarly, safety activists, such as MADD members, have opposed allowing youth peer or “teen” courts, which are used in some communities to handle minor offenses, such as to hear drinking-and-driving cases, because these courts do not have the authority to impose license suspensions.

### Civil Proceedings

DWI offenses are considered criminal offenses and, therefore, are tried in criminal court. Recently, however, several jurisdictions in the State of New York have implemented a policy of confiscating the vehicles of DWI offenders (Safir et al. 2000), which is a civil action and requires a separate judicial process from the criminal trial. This process is managed by the city attorney, rather than the local prosecutor, and requires a lower standard of proof for the sanction to be imposed (i.e., “preponderance of the evidence,” rather than “beyond a reasonable doubt”). Although this policy has yet to be fully evaluated, it has been upheld by the courts and is likely to be implemented in other jurisdictions. It remains to be seen whether such vehicle forfeiture programs can be cost-effective—in some localities the costs for towing and storing a vehicle are greater than its resale value (Voas 1992). If the programs are cost-effective and reduce recidivism, however, a greater proportion of DWI caseloads will flow into civil courts.

### Pretrial Hearings

For DWI cases that are being adjudicated in criminal court, hearings may be held well before the trial begins. These pretrial hearings may result in the imposition of important sanctions, such as license suspension and vehicle impoundment. Because these hearings occur shortly after the offense, they have the important feature of immediacy, which, according to hypotheses, makes them more effective in deterring offenders (Ross 1984). Pretrial hearings may be held for several reasons:

- ALR and suspension laws require that the offender have an opportunity to receive a hearing. Although most of these cases are heard before a DMV hearing officer, in some States the hearings occur in the criminal court.
- Criminal courts may hold bail hearings for offenders who are being held on more serious DWI-related charges, such as vehicular homicide.
- In some jurisdictions, bail hearings are held for DWI offenders, during which installation of an interlock can be imposed as a condition of pretrial release.
- In States where the vehicles driven by DWI offenders are being impounded (e.g., Ohio and New York), the courts are required to hold a hearing on the seizure of the vehicle if the owner of record requests it.

### Pretrial Programs

Some efforts to prevent recidivism among DWI offenders, particularly those drivers who suffer from alcohol-use disorders, focus on motivating the offenders to participate in treatment programs. One way to achieve this goal is by postponing a trial pending the completion of the remedial program, a process called diversion. Successful completion of the treatment or education program, together with a period of driving without a repeat offense, can earn the offender a dismissal of the DWI charge. Safety advocates have strongly opposed such programs, because the offender’s driving record remains clear. As a result, some offenders in States such as Illinois have been apprehended multiple times without ever receiving a DWI conviction. Illinois recently has corrected this record-keeping problem, however, by allowing drivers to receive only one such “court supervision” in a lifetime. Researchers have been unable to assess the effectiveness of diversion programs, because the lack of record keeping makes it difficult to determine the effect of these measures.

Similar to Illinois, the State of Oregon has a diversion program that results in a permanent record that although not a conviction, prevents the offender from receiving a second diversion. An evaluation by the Oregon Traffic Safety Commission found that offenders who elected diversions had lower recidivism rates than those who refused or were denied participation because of their driving records (see Fields 1994). However, whether the reductions can be attributed to the program is unclear, because the offenders were not randomly assigned to the diversion program.

Although most diversion programs are limited to first-time offenders, some diversion programs are open to repeat offenders. Salzberg and Klingberg (1983) evaluated one such program in the State of Washington and found that the participants had higher, rather than lower, recidivism rates than nonparticipating offenders. One possible reason for the higher recidivism rates was that the participants were free to drive, whereas nonparticipants were fully suspended. In Monroe County, New York, second-time DWI cases (which are considered felonies) overwhelmed the criminal justice system to such an extent that in 1980 the prosecutor implemented a pretrial diversion system, which has been in operation since that time (Fields 1994). Under the program, the court adjourns the case for 6 months while the offender undergoes treatment and is closely supervised by a probation officer. At the end of that period, if the offender has performed satisfactorily, the charge is reduced to a misdemeanor DWI (i.e., a first-time offense), otherwise the felony second-offense trial proceeds. An evaluation of this program compared the reoffense rate of offenders who successfully completed the program (i.e., 9 percent) with offenders who were either denied participation (a reoffense rate of 19 percent) or failed to complete the program (a reoffense rate of 16 percent) (Pretrial Services Corporation of the Monroe County Bar Association 1993). However, because this was not a random assignment study, these differences may have been attributable to nonequivalent comparison groups.

Not all pretrial programs allow the offender to avoid a DWI conviction on the driving record through program participation. For example, a pretrial, intensive supervision probation (ISP) program in Milwaukee County, Wisconsin, engages repeat DWI offenders in treatment shortly after arrest and provides ongoing monitoring supervision throughout the pretrial period. However, participation in the program does not avoid the conviction itself but may reduce the penalties. Participants in the ISP program had substantially lower 1-year recidivism rates (i.e., 5.9 percent) than did the comparison group (i.e., 12.5 percent) that received other sanctions, including jail sentences mandated for repeat offenders (Jones et al. 1996).

### Presentencing Programs

Some States and jurisdictions have instituted interventions that are initiated after conviction but before sanctioning and which influence the severity of the final sanction. These presentencing programs usually avoid the driver record problem presented by pretrial programs. In many cases, as in the State of Maryland’s Probation Before Judgement (PBJ) procedure, however, States allow the offense to be struck from the driver’s record after a certain period of offense-free driving. Maryland offers participation in the PBJ program to DWI offenders as an alternative to a DWI conviction. In that program, the judge can attach conditions to the probation, similar to any other probationary sentence. If the offender complies with all conditions during the probationary term (e.g., attends a treatment program and remains abstinent), the conviction is expunged. Because the PBJ intervention is not considered a public record, it does not affect an offender’s automobile insurance or commercial license. In a recent study examining the effectiveness of sanctioning versus rehabilitation approaches, Taxman and Piquero (1998) found that from 1985 through 1993, 65 percent of first-time offenders in Maryland received a PBJ compared with 11 percent of repeat offenders. Analyses on recidivism risk were conducted on the full sample (i.e., both first-time and repeat offenders) as well as on first-time offenders alone. Results for the full sample indicated that several of the rehabilitative sentences (e.g., alcohol education and alcoholism treatment) appeared to reduce the likelihood of recidivism, whereas the various punitive sanctions (e.g., a “guilty” verdict rather than a PBJ disposition, license restriction, jail sentence, probation, and fines) did not significantly alter the likelihood of recidivism. First-time offenders, however, did not appear to respond well to rehabilitation strategies. Instead, their risk of reconviction was reduced if they received a sentence of PBJ rather than a guilty disposition.

Presentencing programs in the adjudication of DWI offenses also can include screening procedures—that is, a triage process of determining which first-time offenders have a significant drinking problem and, therefore, require assignment to an extended treatment program rather than a short alcohol education program. (Screening is much less important for multiple offenders who, by virtue of being apprehended for DWI more than once, have demonstrated that they are likely to have a drinking problem.) A court presentence investigator normally conducts the screening procedure, and the result is provided to the judge at the time of sentencing. The qualifications of these presentence investigators vary. In many cases they are recovering alcoholics, rather than qualified clinicians, who rely heavily on the offender’s driving record and on his or her self-reports of alcohol use and related problems. Several effective questionnaire instruments, such as the Mortimer-Filkins inventory, the CAGE questionnaire, and the MAST, have been developed to assist in this process (Popkin et al. 1988). In some cases, courts may sentence offenders to attend programs administered by a State agency or a private service company and allow that agency or company to screen the drivers after conviction.

### Probation

DWI offenders, like other criminal offenders, typically are unmotivated to comply with the court-imposed sanctions, including those, such as treatment, that are designed to be beneficial and facilitate recovery from alcohol or other drug dependence. The application of the court’s probation authority, which is based on its power to impose a jail sentence, is critical to assuring offender compliance with treatment, education, house arrest, interlock, and other sanction programs. A major problem for the courts, however, is the limited availability of probation officers, which results in limited supervision of most DWI offenders.

Several studies have demonstrated the potential strength of monitoring programs. Reis (1983) found that for repeat DWI offenders, 15-minute biweekly meetings with a probation officer were as effective in reducing DWI recidivism as were weekly group therapy sessions. Similarly, Voas and Tippetts (1990) noted that first-time DWI offenders required to report weekly to alcohol program monitors had two-thirds fewer subsequent DWI offenses compared with offenders without that requirement.

More extensive forms of probation also can reduce recidivism effectively, as shown by the Milwaukee ISP program mentioned previously and the Day Reporting Center (DRC) in Maricopa County, Arizona. The DRC is a highly structured, nonresidential facility providing supervision, reporting, employment, counseling, education, and community resource referrals to probationers convicted of felony DWI. The DRC program provides a continuum of correctional services to augment intensive supervision, residential programs, and regular supervision at a significantly lower cost than that incurred for incarcerating offenders. However, Jones and Lacey (1999) found that the DRC was no more effective in reducing recidivism than was a standard probation program used in the study jurisdiction. One reason for the failure to detect a difference may have been that offenders in both programs had low reconviction rates of about 8 percent after 2 years.

### Extended Supervision by Judges

As judges gain seniority, they frequently choose to adjudicate more serious offenses than DWI, resulting in a high turnover among judges handling DWI cases. A few judges, however, elect to focus on DWI offenders and develop their own special approaches to sentencing DWI offenders. Typically, these programs involve monitoring offenders closely. Examples of such innovative programs are those developed by Judge Culver in Indiana, who gives offenders the alternative of installing an ignition interlock or going to jail, and by Judge Adkins in Ohio, who uses lie detectors to determine whether offenders have complied with the probation requirement for abstinence. Most such initiatives have not been evaluated. However, Jones and Lacey (1998) studied a program designed by Judge Todd in Georgia, who uses his own screening data system, along with a wide variety of traditional and innovative programs, to create an individualized sanctioning program for each offender beyond the minimum mandatory penalties. Offenders receiving this intervention demonstrated a 50-percent reduction in DWI recidivism compared with offenders adjudicated by other judges.

Currently, the best examples of extended judicial monitoring of alcohol and other drug abuse offenders are drug courts. These courts have been developed over the past decade to take nonviolent drug offenders out of traditional court systems and place them in programs designed to promote recovery, reduce recidivism, and save money (Tauber and Huddleston 1999). To this end, drug courts take a rehabilitative approach—characterized by clinical assessment and intensive treatment—to initiate behavioral changes in offenders with clinically significant drug-abuse problems. Drug courts also typically feature close supervision by the judge as well as by the treatment provider, including frequent drug testing and a demand for offender accountability. Approximately one-half of all drug-court programs involve diversion. For offenders who successfully complete the treatment program, the original charges may be reduced or dropped, whereas offenders who fail to complete the program face prosecution and sentencing for the original offense. Currently, approximately 550 drug courts exist or are being planned in the United States.

The application of the drug-court model to DWI cases is based on the notion that the traditional judicial approach (i.e., sanctions) has reduced alcohol-related crashes primarily among social drinkers and drivers under the legal drinking age. Problem drinkers, however, generally are not deterred by traditional sanctions and continue to drink as well as drive after drinking until they are successfully treated for their addiction. Accordingly, DWI court programs are typically for repeat offenders who are most likely to be problem drinkers. In some jurisdictions, certain drug courts operate solely to hear DWI cases (i.e., DWI courts), whereas in other jurisdictions drug courts adjudicate both DWI and other drug cases. Because they are a recent development in the effort to prevent impaired driving, few drug courts handling DWI cases have been evaluated.

## Summary

Based on the research conducted to date, it appears that the two most effective means of reducing recidivism among DWI offenders are rehabilitative programs and incapacitation, whereas the usefulness of punishment (e.g., incarceration and fines) appears limited. Although great variation exists in treatment and education programs and their effectiveness, evidence suggests that when such programs are delivered alone (i.e., without other intervention components), modest reductions in recidivism are attained. Effectiveness is increased, however, when remedial interventions are comprised of several modalities (including contact probation, which has been shown independently to reduce recidivism risk). The strongest evidence for using combination interventions comes from numerous studies showing significant reductions in reconviction rates over several years when treatment and education programs are combined with license suspension. One essential factor in ensuring treatment effectiveness (and in supporting recovery of alcohol-dependent drivers) is monitoring offenders’ participation in such programs.

Restricting offenders’ driving opportunities while treatment interventions take place is a growing problem. Similarly, the increasing number of drivers whose licenses have been suspended as the result of ALR laws has made enforcement of these suspensions difficult. To cope with these problems, an increasing number of States will probably turn to vehicle impoundment and forfeiture. Other strategies that will play an increasing role in incapacitation approaches in the future are technological measures, such as electronic house arrest monitoring and vehicle interlocks. Because these methods of monitoring behavior are intrusive, offenders will strongly resist their application. Consequently, such sanctions generally will need to be imposed by the courts, which have the power to impose incarceration on offenders who fail to comply.

## Footnotes

1 Insurance companies have recognized this “maturing out” of problem behavior for years as evidenced by their higher premiums for youthful male drivers that end at age 25 or when the driver marries.

2 The term “problem drinkers” refers here to those people who abuse alcohol and frequently experience negative consequences, in contrast to “social drinkers,” who occasionally consume sufficient alcohol to impair their driving but are not generally seen as abusing or being dependent on alcohol.

3 Disulfiram is a medication used in alcoholism treatment. When taken with alcohol, disulfiram causes highly unpleasant effects, such as nausea and vomiting. These effects deter alcoholic patients from consuming alcohol while taking the medication.
